# Esaxerenone versus angiotensin II receptor blockers as second-line therapy in older Japanese patients with uncontrolled hypertension on calcium channel blockers: the randomized, open-label ESCORT-HT study

**DOI:** 10.1038/s41440-026-02634-4

**Published:** 2026-04-24

**Authors:** Kazuomi Kario, Hiroyuki Ohbayashi, Hajime Ishii, Mitsutoshi Kato, Minoru Nozaki, Norio Abiru, Toshiki Fukui, Kazushi Nomura, Yasushi Fukushima, Naoki Itabashi, Kazuaki Uchiyama, Masafumi Nishizawa, Yoshiki Hata, Noriko Nakamura, Satoshi Kodono, Kunio Hirano, Tomohiro Katsuya, Tatsuo Shimosawa, Kazuhito Shiosakai, Go Kato, Takashi Taguchi, Mitsuru Ohishi

**Affiliations:** 1 Division of Cardiovascular Medicine, Department of Medicine, Jichi Medical University School of Medicine, Shimotsuke, Japan; 2 Tohno Chuo Clinic, Mizunami, Gifu Japan; 3 Kashinoki Clinic, Date, Fukushima Japan; 4 Kato Clinic of Internal Medicine, Katsushika-ku, Tokyo, Japan; 5 Medical Corporation Shirayurikai Swing Nozaki Clinic, Musashino, Tokyo, Japan; 6 Midori Clinic, Nagasaki, Nagasaki Japan; 7 Olive Takamatsu Medical Clinic, Takamatsu, Kagawa Japan; 8 Nomura Clinic, Itabashi-ku, Tokyo, Japan; 9 Fukuwa Clinic, Chuo-ku, Tokyo Japan; 10 Itabashi Diabetes and Dermatology Medical Clinic, Koga, Ibaraki Japan; 11 Uchiyama Clinic, Joetsu, Niigata Japan; 12 Minamisanriku Hospital, Motoyoshi, Miyagi Japan; 13 Minamino Cardiovascular Hospital, Hachioji, Tokyo Japan; 14 Primula Clinic, Kagoshima, Kagoshima, Japan; 15 Fukuhama Chuo Clinic, Fukuoka, Fukuoka, Japan; 16 Hirano Clinic, Morioka, Iwate Japan; 17 Katsuya Clinic, Amagasaki, Hyogo Japan; 18 Department of Clinical Laboratory, School of Medicine, International University of Health and Welfare, Narita, Chiba Japan; 19 Data Intelligence Department, Daiichi Sankyo Co., Ltd., Shinagawa-ku, Tokyo Japan; 20 Primary Medical Science Department, Medical Affairs Division, Daiichi Sankyo Co., Ltd., Chuo-ku, Tokyo Japan; 21 Department of Cardiovascular Medicine and Hypertension, Graduate School of Medical and Dental Sciences, Kagoshima University, Kagoshima, Kagoshima Japan

**Keywords:** Angiotensin II receptor blockers, Esaxerenone, Mineralocorticoid-receptor blockers, Morning hypertension, Randomized comparative study

## Abstract

Mineralocorticoid receptor blockers (MRBs) are positioned as second-line antihypertensive agents in the 2025 Japanese Society of Hypertension guidelines, yet evidence in older patients remains limited. This 12-week, multicenter, randomized, open-label, parallel-group, non-inferiority ESCORT-HT study (jRCTs031240300; September 2024–June 2025) compared esaxerenone with angiotensin II receptor blockers (ARBs) as add-on therapy to amlodipine in Japanese patients aged ≥65 years whose morning home systolic blood pressure (SBP) remained ≥135 mmHg despite stable amlodipine. The mean age was 75.5 years in both the esaxerenone (*n* = 202; female: 52.5%) and ARB (*n* = 200; female: 56.5%) groups. At the end of treatment, the least squares mean change from baseline in morning home SBP (primary endpoint) was −10.6 (95% confidence interval: −12.0, −9.1) mmHg with esaxerenone treatment and −9.0 (−10.4, −7.5) mmHg with ARB treatment; the between-group difference was −1.6 (−3.7, 0.5) mmHg, meeting the pre-defined non-inferiority margin (3.8 mmHg). Both treatments lowered the urine albumin-to-creatinine ratio, whereas only esaxerenone significantly reduced N-terminal pro-B-type natriuretic peptide. Treatment-emergent adverse events occurred in 25.1% and 30.8% of the esaxerenone and ARB groups; serious events were reported in 2 versus 7 patients, including one death (esaxerenone group). Hyperkalemia occurred in one esaxerenone-treated patient and none who received ARBs. No serious adverse event was judged to be drug-related. Esaxerenone was non-inferior to ARBs in lowering morning home SBP and showed a favorable safety profile in older Japanese patients with inadequately controlled hypertension on amlodipine. These data support the clinical use of esaxerenone as an effective second-line treatment option for this population.

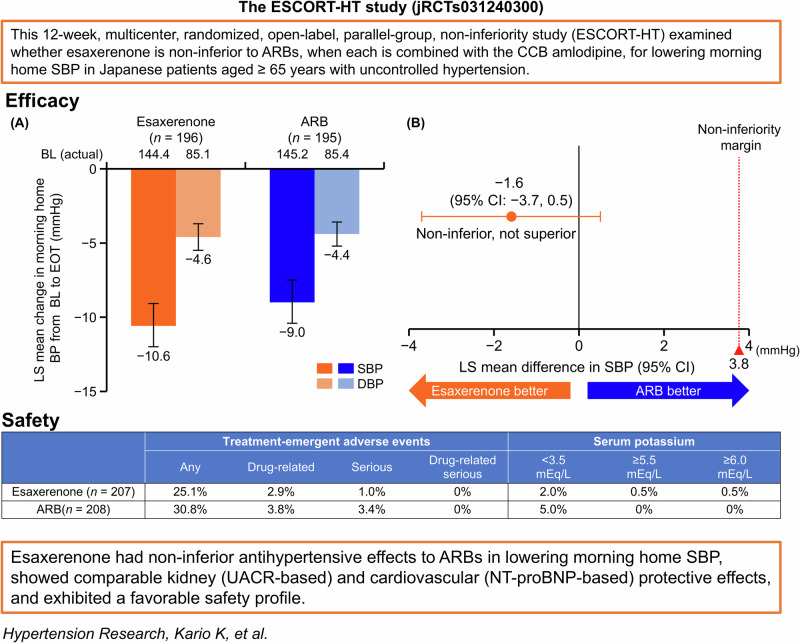

## Introduction

Hypertension is the most prevalent chronic condition in Japan, affecting approximately 43 million individuals [1]. According to the Japanese 2019 National Health and Nutrition Survey, 29.9% of men and 24.9% of women surveyed had a systolic blood pressure (SBP) ≥ 140 mmHg [2, 3]. Despite treatment, an estimated 12.5 million patients in Japan fail to attain guideline-recommended blood pressure (BP) targets [4]. Given that both the prevalence and severity of hypertension increase with age [5] and that Japan is the world’s most rapidly aging society [6], there is an urgent need for effective, age-appropriate strategies to manage hypertension.

The 2025 Japanese Society of Hypertension (JSH 2025) guidelines recommend the use of angiotensin II receptor blockers (ARBs), angiotensin-converting enzyme inhibitors, calcium channel blockers (CCBs), thiazide diuretics, and beta blockers as first-line (step 1 [G1]) treatments for adults of any age [4]. When monotherapy is insufficient, early initiation of combination antihypertensive therapy is recommended to achieve adequate BP control [4]. Notably, mineralocorticoid receptor blockers (MRBs) have been reclassified in the JSH 2025 guidelines from the fourth-line to the preferred second-line (G2) agents, reflecting the increasing recognition of their potent antihypertensive, kidney-protective, and cardioprotective effects. In Japan, an analysis of prescription data found that 38% of treated patients are managed on monotherapy, while 36% require two antihypertensive agents [7]. Among those on dual therapy, CCBs + ARBs account for approximately 60% of all combinations. Thus, MRBs can now be selected as the G2 agents in a substantial proportion of patients.

In routine clinical practice in Japan, ARBs and CCBs (both classified as G1a agents) are the most frequently prescribed first- and second-line combination therapies, and CCBs are the most frequently prescribed therapy in older adults [8]. Age-related declines in renin–angiotensin system activity may attenuate the efficacy of ARBs in this population [9, 10]. In contrast, MRBs effectively lower BP in salt-sensitive or low-renin states, which are common in older patients [11, 12]. However, direct comparative evidence evaluating MRBs versus ARBs as add-on therapy to CCBs in older Japanese patients is limited, and further investigation into their benefits in this population is warranted.

Esaxerenone is a selective, non-steroidal MRB with higher selectivity and potency, a longer half-life, and more favorable bioavailability than other MRBs [13, 14]. In the EXCITE-HT study, esaxerenone added to either an ARB or a CCB demonstrated non-inferior morning home BP reductions versus trichlormethiazide and showed superior efficacy in patients with uncontrolled essential hypertension [15]. Additionally, subgroup analyses based on the type of baseline antihypertensive agent used (ARB or CCB) and patient age (<65 and ≥65 years) showed similar results regardless of the type of baseline antihypertensive agent [16] or patient age [17]. Additionally, the combination of ARB and CCB has been reported to be superior to an ARB plus a diuretic in patients with uncontrolled nocturnal hypertension [18]. However, it remains unclear whether esaxerenone is comparable to ARBs when each is added to CCBs in older patients with uncontrolled hypertension.

Therefore, the ESCORT-HT study was conducted to determine whether esaxerenone is non-inferior to ARBs, when each is combined with the CCB amlodipine, for lowering morning home SBP in Japanese patients aged ≥ 65 years with uncontrolled hypertension. The safety profiles of the two treatment regimens were also compared. Directly comparing the most frequently used G2 agents (CCB + ARB) with the CCB + MRB combination that has been endorsed by the new JSH 2025 guidelines is clinically relevant, especially in older Japanese patients who often receive CCBs as first-line therapy.

## Methods

### Study design

The ESCORT-HT study was a multicenter, randomized, ARB-controlled, open-label, parallel-group study conducted at 53 sites (Supplementary Table 1) between September 2024 and June 2025. Full details of the study design have been published previously [19]. Briefly, patients were randomized in a 1:1 ratio to receive either esaxerenone or an ARB (control), with BP and age used as block allocation variables.

The study protocol was approved by the Certified Review Board of Hattori Clinic (CRB3180027), and written informed consent was obtained from all patients prior to enrollment. The study was conducted in accordance with the principles of the Declaration of Helsinki and the Clinical Trials Act in Japan and was registered in the Japan Registry of Clinical Trials on August 27, 2024 (identifier jRCTs031240300; https://jrct.mhlw.go.jp/en-latest-detail/jRCTs031240300).

### Study participants

Full inclusion and exclusion criteria have been reported previously [19]. Briefly, patients aged ≥ 65 years who had previously received treatment with a CCB (amlodipine 2.5 mg or 5 mg) at the same dose for ≥4 weeks before registration and with a mean morning home SBP ≥ 135 mmHg were included. Patients with secondary hypertension (including endocrine hypertension), hyperkalemia or serum potassium > 4.8 mEq/L, or kidney dysfunction (creatinine-based estimated glomerular filtration rate [eGFR_creat_] < 45 mL/min/1.73 m^2^) were excluded.

### Study interventions

Patients continuously received amlodipine at a constant dose until the end of treatment (EOT). In the esaxerenone group, patients received esaxerenone at a starting dose of 2.5 mg/day, per the package insert [20]. Patients with an eGFR_creat_ 45–59 mL/min/1.73 m^2^ or those with diabetes mellitus and albuminuria at baseline received a starting esaxerenone dose of 1.25 mg/day. The dose of esaxerenone could be increased to 2.5 mg/day at 4 weeks or 8 weeks, as judged by the attending physician, based on the achievement of target BP and serum potassium level (<5.0 mEq/L).

In the ARB group, one ARB at the standard dose (azilsartan, irbesartan, olmesartan medoxomil, candesartan cilexetil, telmisartan, valsartan, or losartan potassium) was administered once daily, in line with the relevant Japanese package insert. The dosing regimen, concomitant treatment, and discontinuation criteria have been reported previously [19].

### Study endpoints

The primary endpoint was the change from baseline in morning home SBP at EOT. Secondary endpoints included: the change from baseline in morning home, bedtime home, and office SBP/DBP at EOT (with values collected up to EOT, using the last observation carried forward if required); achievement rate of target BP levels; change from baseline in urine albumin-to-creatinine ratio (UACR), N-terminal pro-B-type natriuretic peptide (NT-proBNP), plasma aldosterone concentration (PAC), plasma renin activity (PRA), aldosterone–renin ratio (ARR), and urinary biomarkers (sodium, potassium, creatinine, and sodium/potassium ratio) at Week 12.

Safety endpoints were treatment-emergent adverse events (TEAEs; coded by System Organ Class and Preferred Term using the Medical Dictionary for Regulatory Activities, Japanese version 28.0), change from baseline in eGFR_creat_ and serum potassium levels, and the proportion of patients with a serum potassium level ≥5.5 mEq/L and ≥6.0 mEq/L.

Biomarkers (PAC, PRA, ARR, serum NT-proBNP) were measured at a single central laboratory using Good Laboratory Practice-compliant, validated assays. Detailed methods for measuring or calculating BP and serum/urinary biomarkers have been reported previously [19].

### Sample size

As described previously [19], the target sample size was set at 380 (190 per treatment group), which was considered to provide sufficient statistical power (≥80% power, 2.5% one-sided type 1 error probability) to determine the non-inferiority of esaxerenone to that of an ARB regarding lowering of morning home SBP.

### Statistical analysis

The full analysis set (FAS) was used for the primary efficacy analysis, the per-protocol set (PPS) was used for the supplemental analysis, and the safety analysis set was used for the safety analysis. Definitions of the analysis sets have been described previously [19].

The BP-lowering effect of esaxerenone was considered non-inferior to that of ARBs if the upper limit of the two-sided 95% confidence interval (CI) for the difference in SBP change between the esaxerenone group and the ARB group was less than 3.8 mmHg. If the upper limit of the two-sided 95% CI was below 0, esaxerenone was considered superior to ARBs in reducing BP. The justification for the selected non-inferiority margin is provided in the Supplementary Methods.

For the primary endpoint, the least-squares (LS) mean change (95% CI) in morning home SBP and the point estimate of the between-group difference (95% CI) were calculated using an analysis of covariance model, with baseline BP, SBP (≥145 and <145 mmHg), and baseline age (≥75 and <75 years) as covariates. Multiplicity adjustments were only made for the primary endpoint, and the superiority of esaxerenone to ARBs was only tested after confirming the non-inferiority.

Missing BP values at EOT were imputed by the last observation carried forward method using data from 4 weeks onward. For patients with missing data prior to 4 weeks of treatment, data were not imputed.

All statistical analyses had a two-sided significance level of 5% unless otherwise noted and were conducted using SAS version 9.4 or later (SAS Institute Inc., Cary, NC, USA).

## Results

### Patients

Overall, 721 patients were assessed for eligibility during the 4-week run-in period, and 419 eligible patients were randomly allocated to the treatment groups (Supplementary Fig. 1). The FAS included 202 patients in the esaxerenone group and 200 patients in the ARB group. In both groups, most patients completed the 12-week study treatment (191/202 in the esaxerenone group and 192/200 in the ARB group).

Baseline patient characteristics were generally similar between the two groups (Table 1). Mean age was 75.5 years in both groups; the proportion of female patients was 52.5% in the esaxerenone group and 56.5% in the ARB group. In the esaxerenone and ARB groups, 38.1% and 37.5% of patients, respectively, had a BMI ≥ 25 kg/m^2^. Regarding complications, 32.2% of patients in the esaxerenone group and 27.5% of patients in the ARB group had type 2 diabetes mellitus. In the esaxerenone and ARB groups, the mean morning home, bedtime home, and office SBP/DBP were 144.4/85.1 mmHg and 145.2/85.4 mmHg, 135.7/78.4 mmHg and 135.0/78.3 mmHg, and 144.7/78.7 mmHg and 145.6/78.2 mmHg, respectively. Baseline characteristics of the PPS are shown in Supplementary Table 2. Initial and final doses of esaxerenone are described in Table 1 and Supplementary Table 2, and those for ARBs are given in Supplementary Table 3.

**Table 1:** Baseline patient characteristics (full analysis set)

	Esaxerenone *N* = 202	ARB *N* = 200
Age, years	75.5 ± 7.1	75.5 ± 7.0
≥ 75 years	104 (51.5)	106 (53.0)
Female	106 (52.5)	113 (56.5)
BMI, kg/m^2^	24.0 ± 3.4	24.3 ± 3.5
≥ 25	77 (38.1)	75 (37.5)
Smoking history		
Current	12 (5.9)	19 (9.5)
Former	83 (41.1)	55 (27.5)
Never	107 (53.0)	126 (63.0)
Alcohol consumption	80 (39.6)	72 (36.0)
Complication	196 (97.0)	192 (96.0)
T2DM	65 (32.2)	55 (27.5)
Dyslipidemia	104 (51.5)	103 (51.5)
CKD	20 (9.9)	14 (7.0)
Heart failure	27 (13.4)	19 (9.5)
Other	181 (89.6)	177 (88.5)
Duration of hypertension, years	7.9 ± 6.8	6.9 ± 6.3
	*n* = 143	*n* = 147
Morning home SBP, mmHg	144.4 ± 9.4	145.2 ± 10.1
	143.0	143.0
	(125, 172)	(124, 184)
Morning home DBP, mmHg	85.1 ± 8.3	85.4 ± 8.0
	86.0	86.0
	(57, 114)	(65, 109)
	*n* = 201	*n* = 199
Bedtime home SBP, mmHg	135.7 ± 11.5	135.0 ± 12.0
	135.0	135.0
	(104, 168)	(107, 167)
	*n* = 201	*n* = 197
	78.4 ± 7.7	78.3 ± 8.5
Bedtime home DBP, mmHg	79.0	78.0
	(55, 99)	(59, 103)
	*n* = 201	*n* = 197
Office SBP, mmHg	144.7 ± 16.8	145.6 ± 16.8
	144.50	144.00
	(88.5, 188.0)	(106.0, 206.5)
Office DBP, mmHg	78.7 ± 10.8	78.2 ± 10.9
	78.50	77.50
	(43.0, 112.5)	(47.0, 111.0)
PAC (pg/mL; CLEIA test)	35.3 ± 24.5	33.9 ± 26.2
<60 pg/mL	171 (84.7)	168 (84.0)
≥60 pg/mL	31 (15.3)	32 (16.0)
PRA, ng/mL/h	0.74 ± 0.72	0.70 ± 0.76
<1.0 ng/mL/h	150 (74.3)	159 (79.5)
≥1.0 ng/mL/h	52 (25.7)	41 (20.5)
ARR	93.2 ± 102.8	81.1 ± 82.9
	*n* = 198	*n* = 198
<100	134 (66.3)	148 (74.0)
≥100	64 (31.7)	50 (25.0)
Serum NT-proBNP, pg/mL	109.7 ± 207.8	114.8 ± 167.6
	*n* = 198	*n* = 198
<55 pg/mL	82 (40.6)	80 (40.0)
55 to <125 pg/mL	70 (34.7)	63 (31.5)
125 to <400 pg/mL	40 (19.8)	46 (23.0)
≥400 pg/mL	6 (3.0)	9 (4.5)
UACR, mg/g Cr	54.8 ± 127.8	82.7 ± 313.3
	*n* = 201	*n* = 199
<30 mg/g Cr	133 (65.8)	125 (62.5)
30 to <300 mg/g Cr	59 (29.2)	66 (33.0)
≥300 mg/g Cr	9 (4.5)	8 (4.0)
HbA1c	6.1 ± 0.8	6.0 ± 0.8
Serum potassium, mEq/L	4.06 ± 0.37	4.02 ± 0.37
	*n* = 202	*n* = 199
eGFR_creat_, mL/min/1.73 m^2^	69.6 ± 13.3	69.8 ± 15.3
	*n* = 201	*n* = 200
30 to <60	48 (23.8)	50 (25.0)
≥60	153 (75.7)	150 (75.0)
Esaxerenone dose at baseline (initial dose), mg	2.0 ± 0.6	-
1.25	81 (40.1)	-
2.5	121 (59.9)	-
Esaxerenone dose at EOT (last dose), mg	2.3 ± 0.5	-
1.25	32 (15.8)	-
2.5	170 (84.2)	-

Data are mean ± standard deviation, *n* (%), or median (minimum, maximum)

*ARB* angiotensin II receptor blocker, *ARR* aldosterone–renin ratio, *BMI* body mass index, *CKD* chronic kidney disease, *CLEIA* chemiluminescence enzyme immunoassay, *DBP* diastolic blood pressure, *eGFR*_*creat*_ estimated glomerular filtration rate (creatinine-based), *EOT* end of treatment, *HbA1c* glycated hemoglobin*, NT-proBNP* N-terminal pro-B-type natriuretic peptide, *PAC* plasma aldosterone concentration, *PRA* plasma renin activity, *SBP* systolic blood pressure, *T2DM* type 2 diabetes mellitus, *UACR* urine albumin-to-creatinine ratio

### BP-lowering effects

**Fig. 1:**
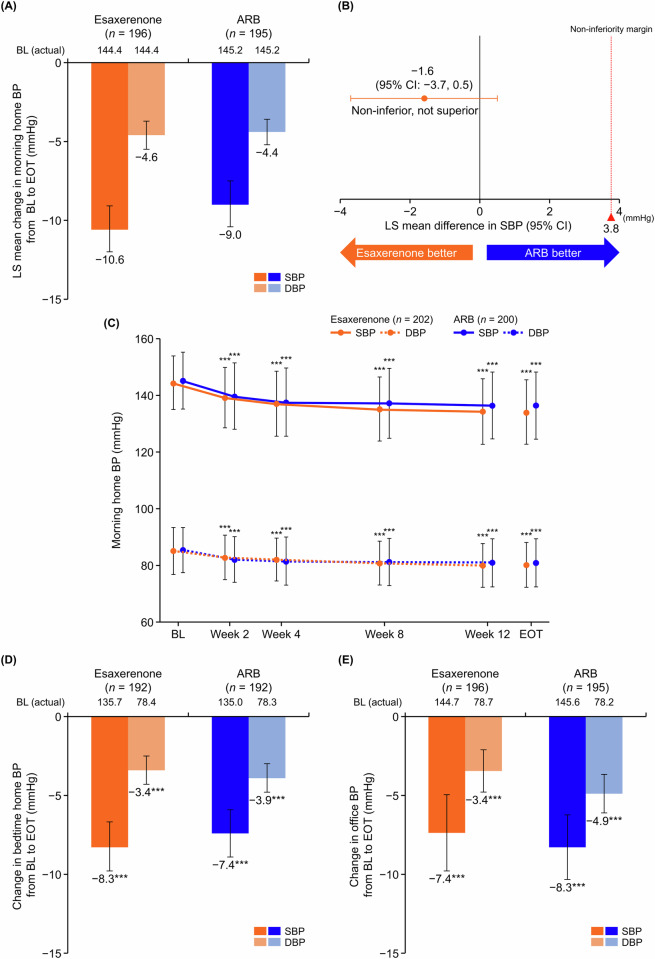
Blood pressure changes with esaxerenone treatment vs ARBs (full analysis set). **A** LS mean change in morning home SBP and mean change in morning home DBP from baseline to EOT. Number of patients for DBP was 194 in each treatment group. Data are shown as means with 95% CI. **B** LS mean difference in morning home SBP reduction. Data at EOT were analyzed using last observation carried forward. **C** Change over time in morning home BP. Data are shown as means with SD. **D** Change in bedtime home BP and **E** office BP from baseline to EOT. Data are shown as means with 95% CI. ***P < 0.001 vs baseline, paired *t*-test. *ARB* angiotensin II receptor blocker, *BL* baseline, *BP* blood pressure, *CI* confidence interval, *DBP* diastolic blood pressure, *EOT* end of treatment, *LS* least squares, *SBP* systolic blood pressure, *SD* standard deviation

The primary endpoint, LS mean change from baseline at EOT in morning home SBP, was −10.6 (95% CI −12.0, −9.1) mmHg in the esaxerenone group and −9.0 (−10.4, −7.5) mmHg in the ARB group (Fig. 1A). The LS mean difference in morning home SBP between the two groups at EOT was −1.6 (−3.7, 0.5) mmHg (Fig. 1B), which was within the pre-defined non-inferiority margin of < 3.8 mmHg. In both groups, morning home SBP/DBP significantly decreased from baseline at Weeks 2 through 12 (all *P* < 0.001) (Fig. 1C). Significant reductions over time were also observed in bedtime home and office SBP/DBP in both treatment groups (all *P* < 0.001) (Fig. 1D, E, and Supplementary Table 4). Similar results were also observed in the PPS (Supplementary Table 4).

The target BP level achievement rates at Week 12 in morning home BP for criteria 1 (SBP/DBP < 135/ < 85 mmHg) were 51.5% (esaxerenone group) and 42.5% (ARB group), and 15.1% (esaxerenone group) and 13.2% (ARB group) for criteria 2 (SBP/DBP < 125/ < 75 mmHg) (Table 2). Similar trends regarding achievement rates were observed for both groups in bedtime home BP and office BP (Table 2), including in the PPS (Supplementary Table 5).

**Table 2:** Achievement rate of target BP levels at Week 12 (full analysis set)

	Criteria 1 Home SBP/DBP, <135/ <85 mmHg Office SBP/DBP, <140/ <90 mmHg		Criteria 2 Home SBP/DBP, <125/ <75 mmHg Office SBP/DBP, <130/ <80 mmHg	
	Esaxerenone *n* = 202	ARB *n* = 200	Esaxerenone *n* = 146	ARB *n* = 152
Morning home BP				
SBP	*n* = 104 51.5% [44.4%, 58.6%]	*n* = 85 42.5% [35.6%, 49.7%]	*n* = 22 15.1% [9.7%, 21.9%]	*n* = 20 13.2% [8.2%, 19.6%]
DBP	*n* = 141 69.8% [63.0%, 76.0%]	*n* = 124 62.0% [54.9%, 68.8%]	*n* = 27 18.5% [12.6%, 25.8%]	*n* = 36 23.7% [17.2%, 31.3%]
Both SBP and DBP	*n* = 93 46.0% [39.0%, 53.2%]	*n* = 71 35.5% [28.9%, 42.6%]	*n* = 8 5.5% [2.4%, 10.5%]	*n* = 10 6.6 [3.2%, 11.8%]
Bedtime home BP				
SBP	*n* = 131 64.9% [57.8%, 71.4%]	*n* = 141 70.5% [63.7%, 76.7%]	*n* = 53 36.3% [28.5%, 44.7%]	*n* = 51 33.6% [26.1%, 41.7%]
DBP	*n* = 175 86.6% [81.2%, 91.0%]	*n* = 170 85.0% [79.3%, 89.6%]	*n* = 64 43.8% [35.6%, 52.3%]	*n* = 71 46.7% [38.6%, 55.0%]
Both SBP and DBP	*n* = 128 63.4% [56.3%, 70.0%]	*n* = 132 66.0% [59.0%, 72.5%]	*n* = 38 26.0% [19.1%, 33.9%]	*n* = 38 25.0% [18.3%, 32.7%]
Office BP				
SBP	*n* = 104 51.5% [44.4%, 58.6%]	*n* = 116 58.0% [50.8%, 64.9%]	*n* = 46 31.5% [24.1%, 39.7%]	*n* = 45 29.6% [22.5%, 37.5%]
DBP	*n* = 173 85.6% [80.0%, 90.2%]	*n* = 177 88.5% [83.2%, 92.6%]	*n* = 88 60.3% [51.9%, 68.3%]	*n* = 97 63.8% [55.6%, 71.4%]
Both SBP and DBP	*n* = 103 51.0% [43.9%, 58.1%]	*n* = 115 57.5% [50.3%, 64.4%]	*n* = 42 28.8% [21.6%, 36.8%]	*n* = 43 28.3% [21.3%, 36.2%]

Data are *n* (%) [95% CI]

Both criteria are based on the 2019 Japanese Society of Hypertension guidelines, and criteria 1 is applied to all patients, but criteria 2 is applied to patients with <75 years of age, chronic kidney dysfunction, or diabetes mellitus

*ARB* angiotensin II receptor blocker, *BP* blood pressure, *CI* confidence interval, *DBP* diastolic blood pressure, *SBP* systolic blood pressure

### Other efficacy endpoints

**Fig. 2:**
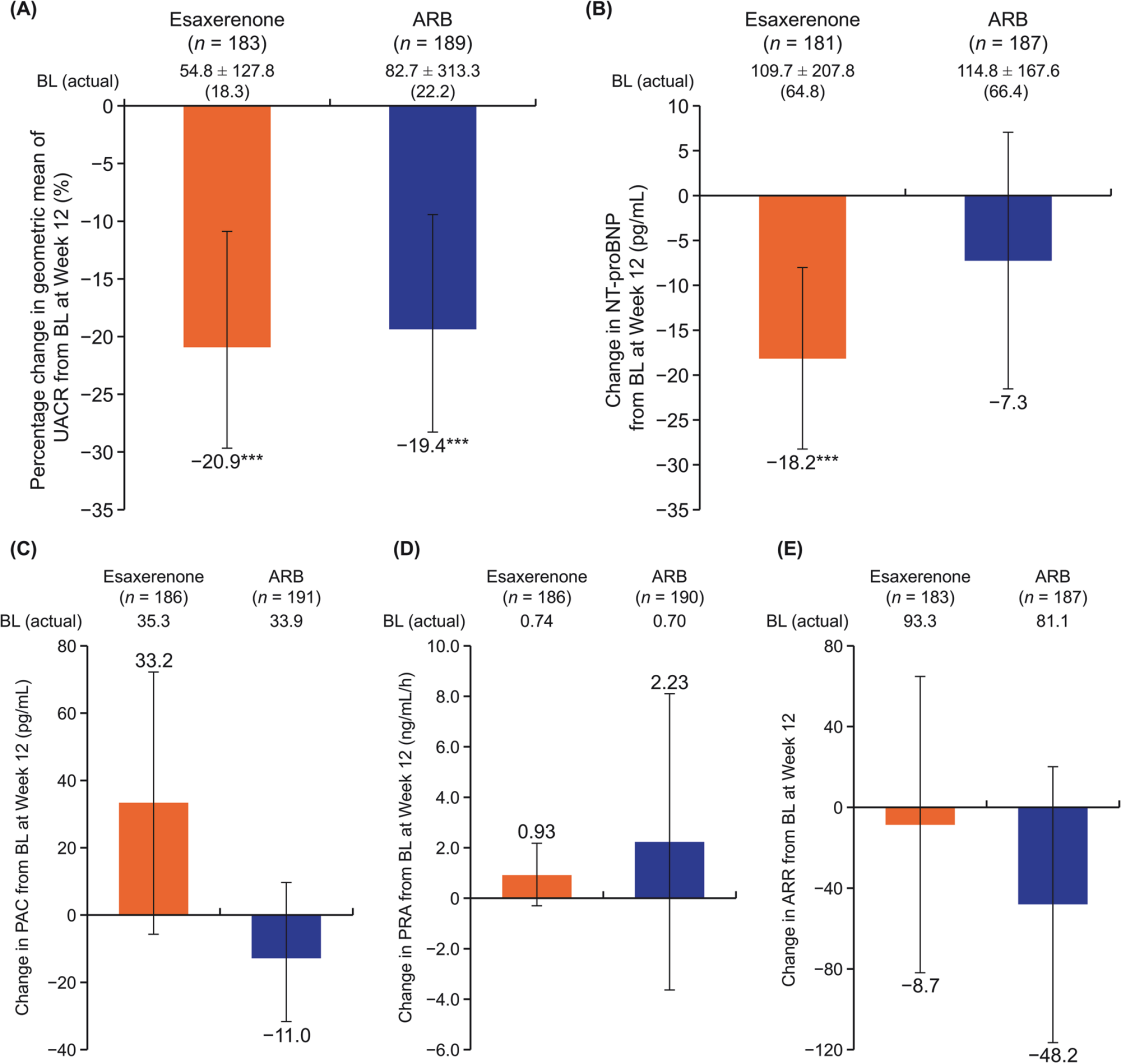
Biomarker results (full analysis set). **A** Percentage change in geometric mean UACR from baseline at Week 12, shown as means with 95% CI. Baseline values are shown as mean ± SD (median). **B** Change in NT-proBNP from baseline at Week 12, shown as means with 95% CI. Baseline values are shown as mean ± SD (median). **C** Change in PAC, **D** PRA, and **E** ARR from baseline at Week 12. Data are shown as means with SD. No significance test was made. ***P < 0.001 vs baseline, paired t-test. *ARB* angiotensin II receptor blocker, *ARR* aldosterone–renin ratio, *BL* baseline, *CI* confidence interval, *EOT* end of treatment, *NT-proBNP* N-terminal pro-B-type natriuretic peptide, *PAC* plasma aldosterone concentration, *PRA* plasma renin activity, *SD* standard deviation, *UACR* urine albumin-creatinine ratio

The geometric mean UACR decreased from baseline at Week 12 in both groups ( − 20.9% for esaxerenone and −19.4% for ARBs, respectively; both *P* < 0.001 versus baseline) (Fig. 2A, Supplementary Table 6). Changes in NT-proBNP levels from baseline at Week 12 were −18.2 (95% CI − 28.3, −8.1; *P* < 0.001) and −7.3 (95% CI − 21.6, 7.0; *P* = 0.313) pg/mL in the esaxerenone and ARB groups, respectively (Fig. 2B and Supplementary Table 6). Similar results were also observed in the PPS (Supplementary Table 6).

PAC and PRA were numerically higher at Week 12 than at baseline in the esaxerenone group (change from baseline at Week 12: 33.2 pg/mL and 0.93 ng/mL/h, respectively) (Fig. 2C, D and Supplementary Table 7). In the ARB group, PAC was numerically lower and PRA numerically higher at Week 12 than at baseline (change from baseline at Week 12: −10.96 pg/mL and 2.23 ng/mL/h, respectively). Changes from baseline at Week 12 in ARR were −8.7 and −48.2 in the esaxerenone and ARB groups, respectively (Fig. 2E). Similar results were also observed in the PPS (Supplementary Table 7).

### Safety

TEAEs were observed in 25.1% of patients with esaxerenone and 30.8% with an ARB (Table 3). Drug-related TEAEs occurred in six patients (2.9%) in the esaxerenone group and eight (3.8%) in the ARB group. TEAEs led to treatment discontinuation in four (1.9%) and two (1.0%) patients, respectively. Serious TEAEs were reported in two (1.0%) and seven (3.4%) patients in the esaxerenone and ARB groups, none of which were considered related to the study drugs. In the esaxerenone group, one patient with comorbid bronchial asthma and interstitial pneumonia developed acute bronchitis due to an infection and died of acute respiratory failure. This death was not considered related to esaxerenone.

**Table 3:** Summary of TEAEs (safety analysis set)

Type of TEAE	Esaxerenone *N* = 207	ARB *N* = 208
Any TEAEs	52 (25.1)	64 (30.8)
Drug-related TEAEs	6 (2.9)	8 (3.8)
Serious TEAEs	2 (1.0)	7 (3.4)
Drug-related serious TEAEs	0 (0.0)	0 (0.0)
Discontinued study treatment due to TEAEs	4 (1.9)	2 (1.0)
Discontinued study treatment due to drug-related TEAEs	2 (1.0)	1 (0.5)
Deaths	1 (0.5)	0 (0.0)
Frequent TEAEs occurring in ≥2 (1%) patients in either treatment group (MedDRA/J preferred terms)		
Nasopharyngitis	12 (5.8)	9 (4.3)
Bronchitis	5 (2.4)	15 (7.2)
Constipation	3 (1.4)	3 (1.4)
Cystitis	3 (1.4)	2 (1.0)
Asthma	3 (1.4)	1 (0.5)
Influenza	2 (1.0)	2 (1.0)
Dizziness	2 (1.0)	2 (1.0)
Upper respiratory tract infection	1 (0.5)	2 (1.0)
Abdominal pain	3 (1.4)	0 (0.0)
Diarrhea	2 (1.0)	1 (0.5)
Contusion	1 (0.5)	2 (1.0)
Nausea	2 (1.0)	0 (0.0)
Arthralgia	0 (0.0)	2 (1.0)
Blood potassium increased	2 (1.0)	0 (0.0)
Renin increased	0 (0.0)	2 (1.0)
Wound	0 (0.0)	2 (1.0)

Data are *n* (%). MedDRA/J version 28.0

*MedDRA/J* Medical Dictionary for Regulatory Activities (Japanese version), *TEAE* treatment-emergent adverse event

The most frequent TEAEs in the esaxerenone group were nasopharyngitis (*n* = 12; 5.8%) and bronchitis (*n* = 5; 2.4%) (Table 3). Bronchitis (*n* = 15; 7.2%) and nasopharyngitis (*n* = 9; 4.3%) were also the most frequent TEAEs in the ARB group.

Regarding serum potassium-related TEAEs, blood potassium increased was reported in two patients (1.0%) in the esaxerenone group. No hyperkalemia, hypokalemia, or blood potassium decreased events were reported. No serum potassium-related TEAEs occurred in the ARB group.

**Fig. 3:**
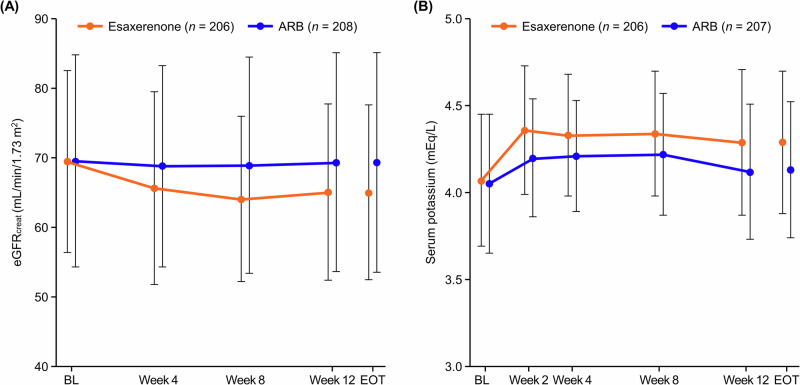
Safety data (safety analysis set). Change in **A** eGFR_creat_ and **B** serum potassium. Data are shown as means with SD. *ARB* angiotensin II receptor blocker, *BL* baseline, *eGFR*_*creat*_ estimated glomerular filtration rate (creatinine-based), *EOT* end of treatment, *SD* standard deviation

In the esaxerenone group, eGFR_creat_ decreased over the first 2 weeks and remained constant until Week 12 (Fig. 3A and Supplementary Table 8). In the ARB group, eGFR_creat_ levels remained consistent throughout the 12-week treatment period.

Serum potassium levels increased over the first 2 weeks after starting treatment, then remained constant up to Week 12 in the esaxerenone group; in the ARB group, serum potassium decreased from Week 8 to Week 12 (Fig. 3B and Supplementary Table 8). The percentages of patients with serum potassium levels <3.5 mEq/L, ≥5.5 mEq/L, and ≥6.0 mEq/L in the esaxerenone group were 2.0%, 0.5%, and 0.5%, respectively (Supplementary Table 9). Both ≥ 5.5 mEq/L and ≥ 6.0 mEq/L categories refer to the same single patient whose peak serum potassium level reached ≥ 6.0 mEq/L. In the ARB group, 5.0% of patients had serum potassium levels < 3.5 mEq/L, and none had serum potassium levels ≥ 5.5 mEq/L or ≥ 6.0 mEq/L.

## Discussion

This study compared the antihypertensive efficacy and safety of esaxerenone versus ARBs in older hypertensive patients with inadequate BP control on CCB monotherapy. Over a 12-week treatment period, this study demonstrated the non-inferiority of esaxerenone to ARBs in reducing morning home SBP, with both groups achieving significant BP reductions. Patients receiving esaxerenone had similar beneficial trends in UACR to those receiving ARBs; however, only patients who received esaxerenone had significantly reduced levels of the heart failure biomarker NT-proBNP. The incidence of TEAEs was comparable between groups, and serum potassium levels and kidney function remained within acceptable ranges. These findings suggest that esaxerenone is an effective therapeutic option for hypertension management in older patients, with antihypertensive effects equivalent to ARBs. This is clinically meaningful because the CCB + ARB approach is currently the predominant dual therapy in Japan, representing about 60% of all two-drug prescriptions [7]. As the CCB + MRB regimen demonstrated comparable BP-lowering efficacy, no clinically significant hyperkalemia, and favorable effects on UACR and NT-proBNP, the results of this study suggest this regimen could be a feasible alternative G2 option for older patients with uncontrolled hypertension. Future studies will be necessary to determine the effects of CCB + esaxerenone treatment on longer-term outcomes, such as cardiovascular and kidney events.

The results of this study are consistent with the EXCITE-HT study [15], which showed that esaxerenone, when added to either an ARB or a CCB, achieved non-inferior morning home BP lowering compared with trichlormethiazide. In the EXCITE-HT study, the mean between-group SBP difference was −2.2 mmHg with a favorable tolerability profile [15], and a subsequent age-stratified analysis showed that esaxerenone was superior to trichlormethiazide in reducing morning systolic BP in patients aged ≥ 65 years (between-group LS mean −3.0 mmHg) [17]. The present study adds to these results by comparing the addition of esaxerenone versus an ARB to ongoing CCB therapy in older patients with uncontrolled hypertension, and demonstrates the non-inferiority of esaxerenone to ARBs. When the results of the EXCITE-HT [15–17] and ESCORT-HT studies are considered together, esaxerenone has shown a consistent antihypertensive effect across different background regimens (including ARBs or CCBs) and patient ages.

A key strength of this study’s design was the use of home BP measurements. Home BP measurements more accurately reflect BP variability in daily life compared with office BP measurements, which are susceptible to “white coat hypertension.” Thus, home BP measurements are considered to provide a more faithful representation of patients’ BP status. Moreover, home BP measurements are better able to predict cardiovascular events than office BP measurements [21–25]. As such, the improvements in home BP observed in this study are more likely to reflect true antihypertensive effects. In contrast to the home BP results, the esaxerenone group showed a tendency towards slightly lower BP reduction in office SBP than the ARB group. This may be explained by the potential sympathetic nervous system inhibitory effects of ARBs in addition to suppression of the renin–angiotensin system. Sympathetic inhibition may be more effective against transient stress responses, as may occur in clinical settings, contributing to greater BP reduction.

Both esaxerenone and ARB treatment showed a beneficial reduction in UACR, suggesting possible kidney-protective effects. This is of particular importance in older populations as kidney function tends to decline with age [26]. Additionally, greater improvements in the biomarker NT-proBNP were observed in the esaxerenone group but not in the ARB group, suggesting that patients treated with esaxerenone had improved cardiac function and a potential reduction in cardiovascular risk [27]. Mineralocorticoid receptor activation has been implicated in myocardial fibrosis, ventricular hypertrophy, and diastolic dysfunction through aldosterone-mediated pathways, independent of BP. Therefore, blockade of these pathways by esaxerenone may exert cardioprotective effects beyond its antihypertensive action. In the present study, the between-group difference in BP reduction was modest and unlikely to fully account for the differential NT-proBNP response, suggesting a BP-independent mechanism. Although heart failure was not listed as an inclusion criterion, a substantial proportion of patients had elevated NT-proBNP at baseline (≥55 pg/mL in approximately 60% of patients), indicating subclinical cardiac stress. It is possible that mineralocorticoid receptor blockade may provide incremental cardioprotection in such patients. These findings are hypothesis-generating and require confirmation in dedicated long-term outcome trials. Nevertheless, as renin–angiotensin system activity reduces with advancing age [28], which may attenuate the efficacy of adding an ARB in older patients with uncontrolled hypertension, our results suggest that targeted mineralocorticoid receptor blockade (such as through esaxerenone treatment) may be an effective alternative for these patients.

The incidence of TEAEs and drug-related TEAEs in the esaxerenone group was comparable to or slightly lower than the ARB group, indicating a favorable safety profile. While serious TEAEs were reported in both groups, including one death in the esaxerenone group, none of these events were considered related to the study drugs. Hyperkalemia is a recognized risk of MRBs [29], but serum potassium levels ≥ 5.5 mEq/L occurred in only one patient in the esaxerenone group, for whom levels were 4.8 mEq/L at baseline, 5.4 mEq/L during Weeks 2–8, and reached 6.3 mEq/L at Week 12. Thus, the risk of electrolyte abnormalities in these older patients remained within acceptable limits. Although eGFR_creat_ had a declining trend during the treatment period, no serious kidney dysfunction was reported, suggesting that esaxerenone did not result in kidney toxicity over the 12-week treatment period. A higher incidence of hypokalemia (serum potassium < 3.5 mEq/L) was reported in the ARB group than in the esaxerenone group (5% vs 2%), which may be attributed to the effect of ARBs on renal sodium retention and potassium excretion. As older patients—particularly those with heart failure—are at higher risk of hypokalemia [30–32], it is important to appropriately manage electrolytes during treatment with ARBs, especially during warmer seasons.

Given the high prevalence of dual therapy and the recent JSH 2025 guideline updates that position MRBs as second-line agents, our data provide evidence to support the wider implementation of CCB + MRB combinations for the management of older Japanese patients with hypertension.

### Limitations

This study had some limitations. As an open-label comparative trial, the potential for bias in the evaluation of treatment efficacy and TEAE reporting cannot be excluded. Awareness of the treatment group may have influenced patients’ adherence to blood pressure measurement protocols or their reporting of TEAEs. However, the use of home BP as the primary endpoint may have partially mitigated observer bias that may have been present with the office BP measurements. Furthermore, the use of a structured measurement schedule and centralized biomarker analysis was designed to minimize subjective variability. As the home BP measurements were collected electronically, the open-label design is not considered likely to have impacted these data. Moreover, safety parameters (including serum potassium levels, hypokalemia, and hyperkalemia) were evaluated separately using clinical laboratory values, allowing an objective assessment of these TEAEs. The treatment duration of 12 weeks was relatively short, necessitating future studies to determine whether the BP-lowering effects observed with CCB and esaxerenone also result in a reduced occurrence of cardiovascular and kidney events. Efficacy and safety data by the type of ARB were not available. As ARBs can exhibit distinct BP-lowering effects depending on the type and dose, this may have affected the observed results. Additionally, higher baseline BP levels may have potentially influenced the choice of each ARB. Only one type of CCB (amlodipine) was used in this study, and the starting dose was limited to 2.5 mg or 5 mg. Whether the observed antihypertensive efficacy and safety profile of esaxerenone as add-on therapy would be maintained with other dihydropyridine CCBs (e.g., azelnidipine or cilnidipine) or at higher amlodipine doses (e.g., 10 mg) remains unknown. Additionally, non-dihydropyridine CCBs were not evaluated. Caution is therefore warranted when extrapolating these findings to clinical settings in which different CCB types or doses are used. Additional studies are required before definitive treatment selection between esaxerenone and ARBs can be generalized to all older patients on CCB monotherapy.

## Conclusion

This study demonstrated the non-inferiority of esaxerenone to ARBs in reducing morning home SBP in older patients with hypertension. Treatment with esaxerenone also improved UACR and heart failure biomarkers, and no unexpected safety concerns were observed. These results indicate that esaxerenone represents an effective therapeutic option with an acceptable safety profile for hypertension management in older patients. Future expansion of the clinical applications of esaxerenone and validation of its long-term benefits are anticipated.

## Supplementary information

Supplementary information

## Data Availability

The anonymized data underlying the results presented in this manuscript may be made available to researchers upon submission of a reasonable request to the corresponding author. The decision to disclose the data will be made by the corresponding author and the funder, Daiichi Sankyo Co., Ltd. Data disclosure can be requested for 36 months from article publication.
