# Supplementary material for: Esaxerenone versus angiotensin II receptor blockers as second-line therapy in older Japanese patients with uncontrolled hypertension on calcium channel blockers: the randomized, open-label ESCORT-HT study
Source: Hypertens Res. 2026 Apr 24;49(7):2240–52. doi: 10.1038/s41440-026-02634-4 (PMC13333488; doi:10.1038/s41440-026-02634-4)
Supplement: Supplementary file 1 — Supplementary information [file 41440_2026_2634_MOESM1_ESM.docx]

**Supplementary Information**

**Supplementary Methods**

For the primary endpoint (change from baseline in morning home systolic blood pressure), the non-inferiority margin was pre-specified as 3.8 mmHg. This value corresponds to 50% of the treatment effect (−7.65 mmHg) that was observed for the reduction in seated systolic blood pressure at Week 8 in a randomized trial that compared irbesartan/amlodipine fixed-dose combination therapy with amlodipine monotherapy in patients with essential hypertension [1]. Retaining 50% of the established angiotensin II receptor blocker effect is consistent with regulatory guidance and has been accepted by the Pharmaceuticals and Medical Devices Agency in Japan. Therefore, the pre-specified margin of 3.8 mmHg ensured that, if non-inferiority was demonstrated, treatment with esaxerenone would preserve a clinically meaningful proportion of the proven benefit of ARB add-on therapy.

**Reference**

1. PMDA Review Report for ‘Aimix® LD/HD’ [irbesartan/amlodipine besylate fixed-dose combination], 2012 [in Japanese], <https://www.pmda.go.jp/drugs/2012/P201200134/40009300_22400AMX01391000_A100_1.pdf>. Accessed 18 December 2025.

**
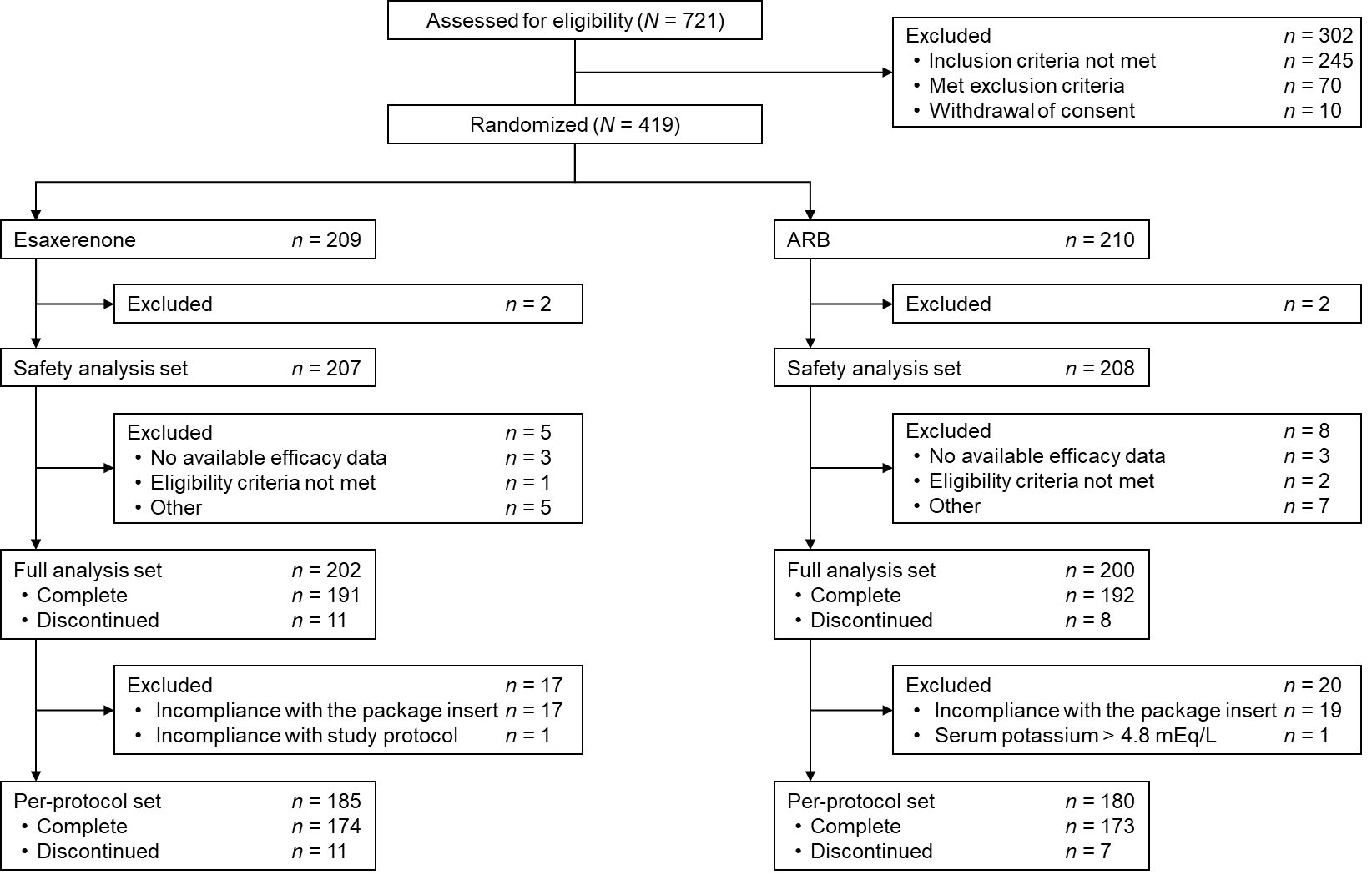
**

**Supplementary Fig. 1.** Patient disposition.

*ARB* angiotensin II receptor blocker.

**Supplementary Table 1.** Participating institutions and principal investigators of ESCORT-HT

| **Institution** | **Principal investigator** |
| --- | --- |
| Jichi Medical University | Kazuomi Kario^1^ |
| Washiya Memorial hospital | Toshimitsu Kitajima |
| Katsuya Clinic | Tomohiro Katsuya |
| Olive Takamatsu Medical Clinic | Toshiki Fukui |
| Kato Clinic of Internal Medicine | Mitsutoshi Kato |
| Medical Corporation Shirayurikai Swing Nozaki Clinic | Minoru Nozaki |
| Hirano Clinic | Kunio Hirano |
| Nishikawa Clinic | Tetsuo Nishikawa |
| Uchiyama Clinic | Kazuaki Uchiyama |
| Medical Corporation Kyoujinkai Clinic Komatsu | Soichi Kurioka |
| Tokyo Center Clinic | Hirotaka Nagashima |
| Hatta Medical Clinic | Tsuguru Hatta |
| Minamino Cardiovascular Hospital | Yoshiki Hata |
| Miyamotonaika Clinic | Takahide Miyamoto |
| Itabashi Diabetes and Dermatology Medical Clinic | Naoki Itabashi |
| Iwase General Hospital | Hiroshi Ohtani |
| Medical corporation Syureikai Tohno Chuo Clinic | Hiroyuki Ohbayashi |
| Nihonbashi Sakura Clinic | Kumie Ito |
| Hashimotojin Clinic | Masami Hashimoto |
| Yamada Clinic | Hiroyuki Yamada |
| Inobe Funai Clinic | Yoshito Inobe |
| Kikuma Clinic | Kenji Yamauchi |
| Kashinoki Clinic | Hajime Ishii |
| Minamisanriku Hospital | Masafumi Nishizawa |
| Ebe Clinic | Yusuke Ebe |
| Primula Clinic | Noriko Nakamura |
| Jiyugaoka Yamada Internal Medicine Clinic | Daishiro Yamada |
| Maeda Clinic | Kazuhisa Maeda |
| Kikuchi Medical Clinic | Hiroshi Kikuchi |
| Nakamura Cardiovascular Clinic | Yuichiro Nakamura |
| Yoshida Clinic | Katsuhiko Yoshida |
| Ishikawa Prefectural Central Hospital | Toshihiko Yasuda |
| Kyoundo Hospital | Katsuaki Okubo |
| Midori Clinic | Norio Abiru |
| Tsuchiura Beryl Clinic | Kota Yamada |
| Southern Tohoku Research Institute for Neuroscience Southern Tohoku Medical Clinic | Masahiro Ono |
| Fukuhama Chuo Clinic | Satoshi Kodono |
| Hisaki Family Clinic | Ryohei Hisaki |
| Morinaga Ueno Clinic | Hiroshi Morinaga |
| Seiwa Clinic | Hideki Tanaka |
| Soyukai Medical Corporation Yamaguchi Clinic, Internal medicine | Tsuyoshi Yamaguchi |
| Koukan Clinic | Takashi Udagawa |
| Musashikoganei Clinic | Hiroyoshi Kanemitsu |
| Kobe City Medical Center West Hospital | Takehiro Nakamura |
| Higashi Takarazuka Satoh Hospital | Hiroto Tamaru |
| Nomura Clinic | Kazushi Nomura |
| Fukuwa Clinic | Yasushi Fukushima |
| Medical Corporation Yamaichi Building Medical Clinic | Toru Arino |
| Shimada Clinic | Kenei Shimada |
| Kouwa Clinic | Tsuyoshi Yamato |
| Tokyo-Eki Center Building Clinic | Arihiro Kiyosue |

^1^Principal investigator of the ESCORT-HT study.

**Supplementary Table 2** Baseline patient characteristics (per-protocol set)

|  | **Esaxerenone**  ***N* = 185** | **ARB**  ***N* = 180** |
| --- | --- | --- |
| Age, years | 75.6 ± 7.2 | 75.2 ± 6.8 |
| ≥ 75 years | 97 (52.4) | 93 (51.7) |
| Female | 100 (54.1) | 97 (53.9) |
| BMI, kg/m^2^ | 24.0 ± 3.5 | 24.5 ± 3.5 |
| ≥ 25 kg/m^2^ | 70 (37.8) | 70 (38.9) |
| Smoking history |  |  |
| Current | 11 (5.9) | 19 (10.6) |
| Former | 75 (40.5) | 52 (28.9) |
| Never | 99 (53.5) | 109 (60.6) |
| Alcohol consumption | 74 (40.0) | 67 (37.2) |
| Complication | 179 (96.8) | 172 (95.6) |
| T2DM | 51 (27.6) | 48 (26.7) |
| Dyslipidemia | 91 (49.2) | 90 (50.0) |
| CKD | 19 (10.3) | 10 (5.6) |
| Heart failure | 25 (13.5) | 18 (10.0) |
| Other | 167 (90.3) | 157 (87.2) |
| Duration of hypertension, years | 8.1 ± 6.8  *n* = 134 | 6.8 ± 6.3  *n* = 129 |
| Morning home SBP, mmHg | 144.5 ± 9.5  144.0  (125, 172) | 145.4 ± 10.0  143.0  (125, 184) |
| Morning home DBP, mmHg | 85.1 ± 8.4  86.0  (57, 114)  *n* = 184 | 85.6 ± 8.1  86.0  (65, 109)  *n* = 179 |
| Bedtime home SBP, mmHg | 135.7 ± 11.7  136.0  (104, 168)  *n* = 184 | 135.3 ± 12.1  136.0  (107, 167)  *n* = 177 |
| Bedtime home DBP, mmHg | 78.3 ± 7.8  79.0  (55, 99)  *n* = 184 | 78.5 ± 8.5  79.0  (59, 103)  *n* = 177 |
| Office SBP, mmHg | 144.8 ± 16.9  145.0  (88.5, 188.0) | 146.1 ± 17.2  145.0  (106.0, 206.5) |
| Office DBP, mmHg | 78.7 ± 11.0  78.5  (43.0, 112.5) | 78.4 ± 11.1  78.0  (47.0, 111.0) |
| PAC (pg/mL; CLEIA test) | 36.1 ± 24.9 | 34.8 ± 26.5 |
| < 60 pg/mL | 155 (83.8) | 149 (82.8) |
| ≥ 60 pg/mL | 30 (16.2) | 31 (17.2) |
| PRA, ng/mL/h | 0.73 ± 0.74 | 0.71 ± 0.78 |
| < 1.0 ng/mL/h | 140 (75.7) | 142 (78.9) |
| ≥ 1.0 ng/mL/h | 45 (24.3) | 38 (21.1) |
| ARR | 97.2 ± 105.4  *n* = 181 | 83.3 ± 85.0  *n* = 178 |
| < 100 | 119 (64.3) | 131 (72.8) |
| ≥ 100 | 62 (33.5) | 47 (26.1) |
| Serum NT-proBNP, pg/mL | 110.5 ± 214.0  *n* = 181 | 114.5 ± 173.0  *n* = 178 |
| < 55 pg/mL | 72 (38.9) | 74 (41.1) |
| 55 to < 125 pg/mL | 66 (35.7) | 56 (31.1) |
| 125 to < 400 pg/mL | 38 (20.5) | 40 (22.2) |
| ≥ 400 pg/mL | 5 (2.7) | 8 (4.4) |
| UACR, mg/g Cr | 45.8 ± 110.2  *n* = 184 | 86.6 ± 329.9  *n* = 179 |
| < 30 mg/g Cr | 129 (69.7) | 115 (63.9) |
| 30 to < 300 mg/g Cr | 49 (26.5) | 56 (31.1) |
| ≥ 300mg/g Cr | 6 (3.2) | 8 (4.4) |
| HbA1c | 6.0 ± 0.8 | 6.0 ± 0.8 |
| Serum potassium, mEq/L | 4.07 ± 0.36  *n* = 185 | 4.03 ± 0.35  *n* = 179 |
| eGFR_creat_, mL/min/1.73 m^2^ | 69.5 ± 13.3  *n* = 184 | 70.4 ± 15.5  *n* = 180 |
| 30 to < 60 mL/min/1.73 m^2^ | 44 (23.8) | 40 (22.2) |
| ≥ 60 mL/min/1.73 m^2^ | 140 (75.7) | 140 (77.8) |
| Esaxerenone dose at baseline (initial dose), mg | 2.0 ± 0.6 | - |
| 1.25 | 81 (43.8) | - |
| 2.5 | 104 (56.2) | - |
| Esaxerenone dose at EOT (last dose), mg | 2.3 ± 0.5 | - |
| 1.25 | 32 (17.3) | - |
| 2.5 | 153 (82.7) | - |

Data are shown as mean ± standard deviation, *n* (%), or median (minimum, maximum).

*ARB* angiotensin II receptor blocker, *ARR* aldosterone–renin ratio, *BMI* body mass index, *CKD* chronic kidney disease, *CLEIA* chemiluminescence enzyme immunoassay, *DBP* diastolic blood pressure, *eGFR_creat_* estimated glomerular filtration rate (creatinine-based), *EOT* end of treatment, *HbA1c* glycated hemoglobin*, NT-proBNP* N-terminal pro-B-type natriuretic peptide, *PAC* plasma aldosterone concentration, *PRA* plasma renin activity, *SBP* systolic blood pressure, *T2DM* type 2 diabetes mellitus, *UACR* urine albumin-to-creatinine ratio.

**Supplementary Table 3** Initial and last doses of ARBs (full analysis set and per-protocol set)

|  | Full analysis set (n = 200) | | Per-protocol set (n = 180) | |
| --- | --- | --- | --- | --- |
|  | Initial dose, mg | Last dose, mg | Initial dose, mg | Last dose, mg |
| Azilsartan | n = 26 | n = 26 | n = 25 | n = 25 |
| Mean ± SD | 17.3 ± 4.5 | 17.7 ± 4.3 | 17.2 ± 4.6 | 17.6 ± 4.4 |
| 10 mg | 7 (3.5) | 6 (3.0) | 7 (3.9) | 6 (3.3) |
| 20 mg | 19 (9.5) | 20 (10.0) | 18 (10.0) | 19 (10.6) |
| Irbesartan | n = 4 | n = 4 | n = 4 | n = 4 |
| Mean ± SD | 50.0 ± 0.0 | 62.5 ± 25.0 | 50.0 ± 0.0 | 62.5 ± 25.0 |
| 50 mg | 4 (2.0) | 3 (1.5) | 4 (2.2) | 3 (1.7) |
| 100 mg | 0 | 1 (0.5) | 0 | 1 (0.6) |
| Olmesartan | n = 25 | n = 25 | n = 25 | n = 25 |
| Mean ± SD | 9.2 ± 1.9 | 14.2 ± 5.3 | 9.2 ± 1.9 | 14.2 ± 5.3 |
| 5 mg | 4 (2.0) | 1 (0.5) | 4 (2.2) | 1 (0.6) |
| 10 mg | 21 (10.5) | 13 (6.5) | 21 (11.7) | 13 (7.2) |
| 20 mg | 0 | 11 (5.5) | 0 | 11 (6.1) |
| Candesartan | n = 75 | n = 75 | n = 70 | n = 70 |
| Mean ± SD | 5.3 ± 2.0 | 5.9 ± 2.2 | 5.5 ± 1.9 | 6.0 ± 2.0 |
| 2 mg | 4 (2.0) | 3 (1.5) | 0 | 0 |
| 4 mg | 45 (22.5) | 35 (17.5) | 44 (24.4) | 35 (19.4) |
| 8 mg | 26 (13.0) | 36 (18.0) | 26 (14.4) | 35 (19.4) |
| 12 mg | 0 | 1 (0.5) | 0 | 0 (0.0) |
| Telmisartan | n = 39 | n = 39 | n = 39 | n = 39 |
| Mean ± SD | 20.5 ± 3.2 | 31.3 ± 10.0 | 20.5 ± 3.2 | 31.3 ± 10.0 |
| 20 mg | 38 (19.0) | 17 (8.5) | 38 (21.1) | 17 (9.4) |
| 40 mg | 1 (0.5) | 22 (11.0) | 1 (0.6) | 22 (12.2) |
| Valsartan | n = 28 | n = 28 | n = 14 | n = 14 |
| Mean ± SD | 42.9 ± 21.6 | 54.3 ± 23.6 | 40.0 ± 0.0 | 57.1 ± 20.5 |
| 20 mg | 8 (4.0) | 4 (2.0) | 0 | 0 |
| 40 mg | 14 (7.0) | 12 (6.0) | 14 (7.8) | 8 (4.4) |
| 80 mg | 6 (3.0) | 12 (6.0) | 0 | 6 (3.3) |
| Losartan | n = 3 | n = 3 | n = 3 | n = 3 |
| Mean ± SD | 25.0 ± 0.0 | 25.0 ± 0.0 | 25.0 ± 0.0 | 25.0 ± 0.0 |
| 25 mg | 3 (1.5) | 3 (1.5) | 3 (1.7) | 3 (1.7) |
| 50 mg | 0 | 0 | 0 | 0 |

Data are mean ± SD or n (%).

*ARB* angiotensin II receptor blocker, *SD*, standard deviation.

**Supplementary Table 4** BP (full analysis set and per-protocol set)

| **Full analysis set** | **Esaxerenone** | | | | **ARB** | | | |
| --- | --- | --- | --- | --- | --- | --- | --- | --- |
| **Morning home BP** | ***n*** | **SBP, mmHg** | ***n*** | **DBP, mmHg** | ***n*** | **SBP, mmHg** | ***n*** | **DBP, mmHg** |
| Baseline | 202 | 144.4 ± 9.4 | 201 | 85.1 ± 8.3 | 200 | 145.2 ± 10.1 | 199 | 85.4 ± 8.0 |
| Week 2 | 196 | 139.2 ± 10.7 | 196 | 82.8 ± 7.8 | 192 | 139.7 ± 11.7 | 192 | 82.1 ± 8.1 |
| Change from baseline | 196 | −5.1 ± 8.2  [−6.2, −3.9]*** | 196 | −2.1 ± 4.4  [−2.7, −1.5]*** | 192 | −5.5 ± 7.4  [−6.6, −4.5]*** | 191 | −3.2 ± 4.3  [−3.8, −2.6]*** |
| Week 4 | 195 | 137.1 ± 11.5 | 194 | 82.1 ± 7.6 | 195 | 137.6 ± 12.0 | 195 | 81.5 ± 8.5 |
| Change from baseline | 195 | −7.1 ± 9.3  [−8.4, −5.8]*** | 194 | −2.7 ± 5.0  [−3.4, −2.0]*** | 195 | −7.5 ± 8.7  [−8.7, −6.3]*** | 194 | −3.8 ± 5.1  [−4.5, −3.1]*** |
| Week 8 | 191 | 135.1 ± 11.3 | 190 | 80.8 ± 7.8 | 192 | 137.2 ± 12.3 | 192 | 81.2 ± 8.3 |
| Change from baseline | 191 | −9.2 ± 10.0  [−10.6, −7.8]*** | 190 | −3.8 ± 5.3  [−4.6, −3.1]*** | 192 | −7.8 ± 9.9  [−9.3, −6.4]*** | 191 | −4.1 ± 5.5  [−4.9, −3.3]*** |
| Week 12 | 191 | 134.3 ± 11.5 | 190 | 80.0 ± 7.8 | 192 | 136.4 ± 11.8 | 192 | 80.9 ± 8.5 |
| Change from baseline | 191 | −9.9 ± 11.1  [−11.5, −8.4]*** | 189 | −4.5 ± 6.1  [−5.4, −3.7]*** | 192 | −8.6 ± 10.5  [−10.1, −7.1]*** | 191 | −4.4 ± 5.8  [−5.3, −3.6]*** |
| LS mean change from baseline [95% CI] | 191 | −10.5  [−12.0, −9.0] | - | - | 192 | −8.9  [−10.3, −7.4] | - | - |
| Difference in LS mean change from baseline [95% CI] | - | −1.6  [−3.7, 0.5] | - | - | - | - | - | - |
| EOT | 196 | 134.1 ± 11.4 | 195 | 80.1 ± 7.9 | 195 | 136.4 ± 11.9 | 195 | 80.9 ± 8.5 |
| Change from baseline | 196 | −10.1 ± 11.2  [−11.6, −8.5]*** | 194 | −4.6 ± 6.3  [−5.5, −3.7]*** | 195 | −8.7 ± 10.5  [−10.2, −7.2]*** | 194 | −4.4 ± 5.8  [−5.2, −3.6]*** |
| LS mean change from baseline [95% CI] | 196 | −10.6  [−12.0, −9.1] | - | - | 195 | −9.0  [−10.4, −7.5] | - | - |
| Difference in LS mean change from baseline [95% CI] | - | −1.6  [−3.7, 0.5] | - | - | - | - | - | - |
| **Bedtime home BP** | ***n*** | **SBP, mmHg** | ***n*** | **DBP, mmHg** | ***n*** | **SBP, mmHg** | ***n*** | **DBP, mmHg** |
| Baseline | 201 | 135.7 ± 11.5 | 201 | 78.4 ± 7.7 | 197 | 135.0 ± 12.0 | 197 | 78.3 ± 8.5 |
| Week 2 | 191 | 131.5 ± 11.1 | 191 | 76.9 ± 7.5 | 191 | 130.6 ± 12.6 | 191 | 75.6 ± 9.0 |
| Change from baseline | 191 | −4.4 ± 8.2  [−5.6, −3.2]*** | 191 | −1.5 ± 4.6  [−2.1, −0.8]*** | 188 | −4.3 ± 8.2  [−5.5, −3.1]*** | 188 | −2.6 ± 4.7  [−3.2, −1.9]*** |
| Week 4 | 191 | 130.1 ± 11.9 | 191 | 76.2 ± 7.7 | 195 | 129.4 ± 12.4 | 195 | 75.3 ± 8.9 |
| Change from baseline | 191 | −5.8 ± 9.3  [−7.2, −4.5]*** | 191 | −2.1 ± 5.4  [−2.9, −1.3]*** | 192 | −5.6 ± 9.4  [−7.0, −4.3]*** | 192 | −2.9 ± 5.4  [−3.6, −2.1]*** |
| Week 8 | 187 | 128.2 ± 11.3 | 187 | 75.0 ± 7.7 | 192 | 128.3 ± 12.8 | 192 | 74.5 ± 8.9 |
| Change from baseline | 187 | −7.8 ± 9.7  [−9.2, −6.4]*** | 187 | −3.2 ± 5.4  [−3.9, −2.4]*** | 189 | −6.8 ± 10.8  [−8.4, −5.3]*** | 189 | −3.7 ± 5.9  [−4.5, −2.8]*** |
| Week 12 | 188 | 127.7 ± 11.3 | 188 | 74.7 ± 7.8 | 192 | 127.6 ± 12.6 | 192 | 74.2 ± 8.8 |
| Change from baseline | 187 | −8.1 ± 10.9  [−9.7, −6.6]*** | 187 | −3.4 ± 6.1  [−4.3, −2.5]*** | 189 | −7.3 ± 10.5  [−8.8, −5.8]*** | 189 | −3.9 ± 6.1  [−4.8, −3.0]*** |
| EOT | 193 | 127.6 ± 11.2 | 193 | 74.8 ± 7.8 | 195 | 127.7 ± 12.5 | 195 | 74.3 ± 8.8 |
| Change from baseline | 192 | −8.3 ± 11.0  [−9.8, −6.7]*** | 192 | −3.4 ± 6.1  [−4.3, −2.5]*** | 192 | −7.4 ± 10.5  [−8.9, −5.9]*** | 192 | −3.9 ± 6.1  [−4.8, −3.0]*** |
| **Office BP** | ***n*** | **SBP, mmHg** | ***n*** | **DBP, mmHg** | ***n*** | **SBP, mmHg** | ***n*** | **DBP, mmHg** |
| Baseline | 202 | 144.7 ± 16.8 | 202 | 78.7 ± 10.8 | 200 | 145.6 ± 16.8 | 200 | 78.2 ± 10.9 |
| Week 2 | 200 | 139.4 ± 17.1 | 200 | 76.3 ± 11.6 | 194 | 138.4 ± 16.9 | 194 | 74.4 ± 11.6 |
| Change from baseline | 200 | −5.4 ± 14.5  [−7.4, −3.4]*** | 200 | −2.4 ± 9.4  [−3.7, −1.1]*** | 194 | −7.2 ± 14.2  [−9.2, −5.2]*** | 194 | −3.8 ± 9.1  [−5.1, −2.5]*** |
| Week 4 | 198 | 138.2 ± 16.3 | 198 | 75.7 ± 11.7 | 195 | 137.2 ± 17.1 | 195 | 74.5 ± 10.6 |
| Change from baseline | 198 | −6.3 ± 15.7  [−8.5, −4.1]*** | 198 | −2.8 ± 10.6  [−4.3, −1.3]*** | 195 | −8.2 ± 14.8  [−10.3, −6.1]*** | 195 | −3.6 ± 9.1  [−4.9, −2.3]*** |
| Week 8 | 192 | 136.8 ± 16.3 | 192 | 74.2 ± 10.4 | 191 | 135.9 ± 17.0 | 191 | 73.6 ± 11.2 |
| Change from baseline | 192 | −8.0 ± 14.3  [−10.1, −6.0]*** | 192 | −4.2 ± 9.2  [−5.5, −2.9]*** | 191 | −9.3 ± 14.2  [−11.3, −7.2]*** | 191 | −4.6 ± 8.8  [−5.8, −3.3]*** |
| Week 12 | 186 | 137.8 ± 16.4 | 186 | 75.1 ± 10.2 | 191 | 137.0 ± 17.4 | 191 | 73.2 ± 11.3 |
| Change from baseline | 186 | −6.9 ± 17.0  [−9.4, −4.5]*** | 186 | −3.1 ± 9.3  [−4.5, −1.8]*** | 191 | −8.1 ± 14.6  [−10.2, −6.0]*** | 191 | −5.0 ± 8.7  [−6.3, −3.8]*** |
| EOT | 196 | 137.2 ± 16.5 | 196 | 75.0 ± 10.1 | 195 | 137.1 ± 17.3 | 195 | 73.2 ± 11.3 |
| Change from baseline | 196 | −7.4 ± 17.2  [−9.8, −5.0]*** | 196 | −3.4 ± 9.5  [−4.8, −2.1]*** | 195 | −8.3 ± 14.6  [−10.4, −6.2]*** | 195 | −4.9 ± 8.7  [−6.1, −3.7]*** |
| **Per protocol set** | **Esaxerenone** | | | | **ARB** | | | |
| **Morning home BP** | ***n*** | **SBP, mmHg** | ***n*** | **DBP, mmHg** | ***n*** | **SBP, mmHg** | ***n*** | **DBP, mmHg** |
| Baseline | 185 | 144.5 ± 9.5 | 184 | 85.1 ± 8.4 | 180 | 145.4 ± 10.0 | 179 | 85.6 ± 8.1 |
| Week 2 | 180 | 139.4 ± 10.6 | 180 | 82.9 ± 7.9 | 174 | 139.6 ± 11.5 | 174 | 82.2 ± 8.2 |
| Change from baseline | 180 | −5.0 ± 8.1  [−6.2, −3.8]*** | 180 | −2.0 ± 4.3  [−2.6, −1.4]*** | 174 | −5.9 ± 7.3  [−7.0, −4.8]*** | 173 | −3.3 ± 4.2  [−3.9, −2.7]*** |
| Week 4 | 178 | 137.5 ± 11.6 | 178 | 82.3 ± 7.6 | 176 | 137.3 ± 11.8 | 176 | 81.4 ± 8.6 |
| Change from baseline | 178 | −6.8 ± 9.4  [−8.2, −5.4]*** | 178 | −2.5 ± 5.0  [−3.2, −1.8]*** | 176 | −8.1 ± 8.8  [−9.4, −6.8]*** | 175 | −4.1 ± 5.1  [−4.8, −3.3]*** |
| Week 8 | 174 | 135.2 ± 11.4 | 174 | 80.9 ± 7.8 | 173 | 137.3 ± 12.1 | 173 | 81.4 ± 8.3 |
| Change from baseline | 174 | −9.2 ± 9.9  [−10.7, −7.7]*** | 174 | −3.8 ± 5.3  [−4.6, −3.0]*** | 173 | −8.0 ± 10.0  [−9.5, −6.5]*** | 172 | −4.2 ± 5.5  [−5.0, −3.3]*** |
| Week 12 | 174 | 134.5 ± 11.5 | 174 | 80.1 ± 7.9 | 173 | 136.5 ± 11.8 | 173 | 80.8 ± 8.6 |
| Change from baseline | 174 | −9.8 ± 10.9  [−11.5, −8.2]*** | 173 | −4.4 ± 6.1  [−5.4, −3.5]*** | 173 | −8.8 ± 10.7  [−10.4, −7.2]*** | 172 | −4.7 ± 5.9  [−5.6, −3.8]*** |
| LS mean change from baseline [95% CI] | 174 | −10.4  [−11.9, −8.8] | - | - | 173 | −9.0  [−10.5, −7.4] | - | - |
| Difference in LS mean change from baseline [95% CI] | - | −1.4  [−3.6, 0.8] | - | - | - | - | - | - |
| EOT | 179 | 134.4 ± 11.4 | 179 | 80.2 ± 8.0 | 176 | 136.4 ± 11.8 | 176 | 80.9 ± 8.5 |
| Change from baseline | 179 | −9.9 ± 11.0  [−11.6, −8.3]*** | 178 | −4.5 ± 6.3  [−5.5, −3.6]*** | 176 | −8.9 ± 10.7  [−10.5, −7.3]*** | 175 | −4.7 ± 5.9  [−5.5, −3.8]*** |
| LS mean change from baseline [95% CI] | 179 | −10.5  [−12.0, −8.9] | - | - | 176 | −9.1  [−10.7, −7.6] | - | - |
| Difference in LS mean change from baseline [95% CI] | - | −1.4  [−3.5, 0.8] | - | - | - | - | - | - |
| **Bedtime home BP** | ***n*** | **SBP, mmHg** | ***n*** | **DBP, mmHg** | ***n*** | **SBP, mmHg** | ***n*** | **DBP, mmHg** |
| Baseline | 184 | 135.7 ± 11.7 | 184 | 78.3 ± 7.8 | 177 | 135.3 ± 12.1 | 177 | 78.5 ± 8.5 |
| Week 2 | 175 | 131.6 ± 10.9 | 175 | 76.9 ± 7.4 | 173 | 130.5 ± 12.6 | 173 | 75.7 ± 9.0 |
| Change from baseline | 175 | −4.3 ± 8.1  [−5.6, −3.1]*** | 175 | −1.3 ± 4.6  [−2.0, −0.6]*** | 170 | −4.8 ± 8.0  [−6.0, −3.6]*** | 170 | −2.7 ± 4.6  [−3.4, −2.0]*** |
| Week 4 | 175 | 130.1 ± 11.8 | 175 | 76.2 ± 7.7 | 176 | 129.5 ± 12.5 | 176 | 75.4 ± 8.8 |
| Change from baseline | 175 | −5.8 ± 9.4  [−7.2, −4.4]*** | 175 | −1.9 ± 5.5  [−2.7, −1.1]*** | 173 | −5.9 ± 9.2  [−7.3, −4.6]*** | 173 | −2.9 ± 5.2  [−3.7, −2.2]*** |
| Week 8 | 171 | 128.0 ± 11.1 | 171 | 74.9 ± 7.6 | 173 | 128.6 ± 13.0 | 173 | 74.7 ± 8.8 |
| Change from baseline | 171 | −8.0 ± 9.8  [−9.5, −6.5]*** | 171 | −3.1 ± 5.5  [−4.0, −2.3]*** | 170 | −6.9 ± 10.8  [−8.5, −5.2]*** | 170 | −3.7 ± 5.9  [−4.6, −2.8]*** |
| Week 12 | 172 | 128.0 ± 11.2 | 172 | 74.8 ± 7.7 | 173 | 127.8 ± 12.7 | 173 | 74.3 ± 8.8 |
| Change from baseline | 171 | −7.8 ± 10.6  [−9.4, −6.2]*** | 171 | −3.2 ± 5.9  [−4.1, −2.3]*** | 170 | −7.4 ± 10.5  [−9.0, −5.8]*** | 170 | −4.0 ± 6.2  [−4.9, −3.0]*** |
| EOT | 177 | 127.8 ± 11.1 | 177 | 74.9 ± 7.7 | 176 | 127.9 ± 12.6 | 176 | 74.4 ± 8.7 |
| Change from baseline | 176 | −8.0 ± 10.8  [−9.6, −6.4]*** | 176 | −3.2 ± 6.0  [−4.1, −2.3]*** | 173 | −7.5 ± 10.6  [−9.1, −5.9]*** | 173 | −4.0 ± 6.2  [−4.9, −3.0]*** |
| **Office BP** | ***n*** | **SBP, mmHg** | ***n*** | **DBP, mmHg** | ***n*** | **SBP, mmHg** | ***n*** | **DBP, mmHg** |
| Baseline | 185 | 144.8 ± 16.9 | 185 | 78.7 ± 11.0 | 180 | 146.1 ± 17.2 | 180 | 78.4 ± 11.1 |
| Week 2 | 183 | 139.7 ± 16.9 | 183 | 76.4 ± 11.8 | 176 | 138.9 ± 17.3 | 176 | 74.7 ± 11.8 |
| Change from baseline | 183 | −5.3 ± 14.7  [−7.4, −3.1]*** | 183 | −2.3 ± 9.5  [−3.7, −0.9]** | 176 | −7.2 ± 14.5  [−9.3, −5.0]*** | 176 | −3.8 ± 9.2  [−5.2, −2.4]*** |
| Week 4 | 181 | 138.5 ± 16.0 | 181 | 75.6 ± 11.9 | 176 | 138.2 ± 17.2 | 176 | 74.9 ± 10.7 |
| Change from baseline | 181 | −6.2 ± 15.9  [−8.5, −3.8]*** | 181 | −2.9 ± 11.0  [−4.6, −1.3]*** | 176 | −7.8 ± 14.8  [−10.0, −5.6]*** | 176 | −3.5 ± 9.2  [−4.8, −2.1]*** |
| Week 8 | 175 | 136.9 ± 16.4 | 175 | 74.2 ± 10.5 | 172 | 136.5 ± 17.4 | 172 | 74.0 ± 11.3 |
| Change from baseline | 175 | −8.1 ± 14.5  [−10.3, −6.0]*** | 175 | −4.2 ± 9.3  [−5.6, −2.8]*** | 172 | −9.2 ± 14.1  [−11.4, −7.1]*** | 172 | −4.5 ± 8.7  [−5.8, −3.2]*** |
| Week 12 | 171 | 138.1 ± 16.2 | 171 | 75.0 ± 10.1 | 172 | 137.5 ± 17.7 | 172 | 73.6 ± 11.2 |
| Change from baseline | 171 | −6.8 ± 17.4  [−9.5, −4.2]*** | 171 | −3.2 ± 9.5  [−4.6, −1.8]*** | 172 | −8.2 ± 14.5  [−10.4, −6.0]*** | 172 | −5.0 ± 8.3  [−6.2, −3.7]*** |
| EOT | 179 | 137.6 ± 16.4 | 179 | 75.1 ± 10.0 | 176 | 137.6 ± 17.6 | 176 | 73.6 ± 11.1 |
| Change from baseline | 179 | −7.1 ± 17.7  [−9.7, −4.5]*** | 179 | −3.4 ± 9.7  [−4.9, −2.0]*** | 176 | −8.4 ± 14.5  [−10.6, −6.2]*** | 176 | −4.8 ± 8.3  [−6.1, −3.6]*** |

Data are mean ± SD [95% CI] unless otherwise specified.

***P* <0.01, ****P* <0.001 versus baseline, paired *t*-test.

LS mean change were calculated for morning home SBP at Week 12 and EOT.

LS mean change (95% CI) in morning home SBP and the point estimate of the between-group difference (95% CI) were calculated using an analysis of covariance model with baseline BP, SBP (≥ 145 and < 145 mmHg), and baseline age (≥ 75 and < 75 years) as covariates.

*BP* blood pressure, *CI* confidence interval, *DBP* diastolic blood pressure, *EOT* end of treatment, *LS* least squares, *SBP* systolic blood pressure, *SD* standard deviation.

**Supplementary Table 5** Achievement rate of target BP levels at Week 12 (per-protocol set)

|  | **Criteria 1**  **Home SBP/DBP, < 135/< 85 mmHg**  **Office SBP/DBP, < 140/< 90 mmHg** | | **Criteria 2**  **Home SBP/DBP, < 125/< 75 mmHg**  **Office SBP/DBP, < 130/< 80 mmHg** | |
| --- | --- | --- | --- | --- |
|  | **Esaxerenone**  ***n* = 185** | **ARB**  ***n* = 180** | **Esaxerenone**  ***n* = 130** | **ARB**  ***n* = 134** |
| Morning home BP |  |  |  |  |
| SBP | *n* = 93  50.3% [42.8%, 57.7%] | *n* = 78  43.3% [36.0%, 50.9%] | *n* = 19  14.6% [9.0%, 21.9%] | *n* = 16  11.9% [7.0%, 18.7%] |
| DBP | *n* = 130  70.3% [63.1%, 76.8%] | *n* = 112  62.2% [54.7%, 69.3%] | *n* = 23  17.7% [11.6%, 25.4%] | *n* = 32  23.9% [16.9%, 32.0%] |
| Both SBP and DBP | *n* = 86  46.5% [39.1%, 54.0%] | *n* = 65  36.1% [29.1%, 43.6%] | *n* = 7  5.4% [2.2%, 10.8%] | *n* = 8  6.0% [2.6%, 11.4%] |
| Bedtime home BP |  |  |  |  |
| SBP | *n* = 118  63.8% [56.4%, 70.7%] | *n* = 125  69.4% [62.2%, 76.1%] | *n* = 47  36.2% [27.9%, 45.0%] | *n* = 44  32.8% [25.0%, 41.5%] |
| DBP | *n* = 161  87.0% [81.3%, 91.5%] | *n* = 153  85.0% [78.9%, 89.9%] | *n* = 55  42.3% [33.7%, 51.3%] | *n* = 62  46.3% [37.6%, 55.1%] |
| Both SBP and DBP | *n* = 117  63.2% [55.9%, 70.2%] | *n* = 116  64.4% [57.0%, 71.4%] | *n* = 34  26.2% [18.8%, 34.6%] | *n* = 34  25.4% [18.3%, 33.6%] |
| Office BP |  |  |  |  |
| SBP | *n* = 94  50.8% [43.4%, 58.2%] | *n* = 102  56.7% [49.1%, 64.0%] | *n* = 41  31.5% [23.7%, 40.3%] | *n* = 40  29.9% [22.3%, 38.4%] |
| DBP | *n* = 159  85.9% [80.1%, 90.6%] | *n* = 158  87.8% [82.1%, 92.2%] | *n* = 79  60.8% [51.8%, 69.2%] | *n* = 85  63.4% [54.7%, 71.6%] |
| Both SBP and DBP | *n* = 94  50.8% [43.4%, 58.2%] | *n* = 101  56.1% [48.5%, 63.5%] | *n* = 37  28.5% [20.9%, 37.0%] | *n* = 38  28.4% [20.9%, 36.8%] |

Data are *n* (%) [95% CI].

Both criteria are based on the 2019 Japanese Society of Hypertension guidelines, and criteria 1 is applied to all patients, but criteria 2 is applied to patients with <75 years of age, chronic kidney dysfunction, or diabetes mellitus.

*ARB* angiotensin II receptor blocker, *BP* blood pressure, *CI* confidence interval, *DBP* diastolic blood pressure, *SBP* systolic blood pressure.

**Supplementary Table 6** UACR and NT-proBNP (full analysis set and per-protocol set)

|  | **Full analysis set** | | **Per-protocol set** | |
| --- | --- | --- | --- | --- |
|  | **Esaxerenone** | **ARBs** | **Esaxerenone** | **ARBs** |
| **UACR, mg/g Cr** |  |  |  |  |
| Baseline | *n* = 201  54.8 ± 127.8 | *n* = 199  82.7 ± 313.3 | *n* = 184  45.8 ± 110.2 | *n* = 179  86.6 ± 329.9 |
| Week 4 | *n* = 197  43.3 ± 111.0 | *n* = 194  75.4 ± 306.5 | *n* = 180  40.7 ± 112.5 | *n* = 175  75.6 ± 320.5 |
| Change from baseline | *n* = 196  −10.0 ± 76.6 | *n* = 193  −6.5 ± 97.1 | *n* = 179  −3.1 ± 60.9 | *n* = 174  −9.9 ± 93.6 |
| Percentage change in geometric mean from baseline [95% CI] | −15.6  [−24.9, −5.2]** | −16.4  [−25.0, −6.9]** | −11.5  [−21.6, −0.1]* | −19.1  [−27.3, −10.0]*** |
| Week 8 | *n* = 191  39.4 ± 90.5 | *n* = 191  75.7 ± 380.4 | *n* = 174  35.6 ± 78.9 | *n* = 172  79.0 ± 400.3 |
| Change from baseline | *n* = 190  −14.8 ± 75.0** | *n* = 190  −7.4 ± 173.6 | *n* = 173  −8.9 ± 60.8 | *n* = 171  −7.8 ± 181.0 |
| Percentage change in geometric mean from baseline [95% CI] | −18.8  [−27.2, −9.6]*** | −21.7  [−29.9, −12.5]*** | −15.1  [−24.0, −5.1]** | −22.6  [−30.4, −14.0]*** |
| Week 12 | *n* = 183  53.2 ± 167.2 | *n* = 190  73.8 ± 276.1 | *n* = 168  49.3 ± 167.9 | *n* = 171  78.7 ± 290.5 |
| Change from baseline | *n* = 183  −2.5 ± 99.2 | *n* = 189  −9.7 ± 100.4 | *n* = 168  3.5 ± 93.0 | *n* = 170  −8.5 ± 104.6 |
| Percentage change in geometric mean from baseline [95% CI] | −20.9  [−29.7, −10.9]*** | −19.4  [−28.3, −9.4]*** | −17.8  [−27.1, −7.2]** | −18.0  [−27.5, −7.3]** |
| **NT-proBNP, pg/mL** |  |  |  |  |
| Baseline | *n* = 198  109.7 ± 207.8 | *n* = 198  114.8 ± 167.6 | *n* = 181  110.5 ± 214.0 | *n* = 178  114.5 ± 173.0 |
| Week 12 | *n* = 184  89.2 ± 165.1 | *n* = 188  108.1 ± 140.0 | *n* = 169  89.0 ± 166.3 | *n* = 169  108.5 ± 143.0 |
| Change from baseline [95% CI] | *n* = 181  −18.2 ± 68.8  [−28.3, −8.1]*** | *n* = 187  −7.3 ± 98.9  [−21.6, 7.0] | *n* = 166  −18.9 ± 70.5  [−29.7, −8.1]*** | *n* = 168  −6.8 ± 103.5  [−22.6, 9.0] |
| Percentage change in geometric mean from baseline [95% CI] | −16.7  [−22.4, −10.7]*** | −6.5  [−13.1, 0.6] | −16.8  [−22.8, −10.3]*** | −6.0  [−13.1, 1.7] |

* *P* < 0.05, ***P* <0.01, ****P* <0.001 versus baseline, paired *t*-test.

Data are mean ± standard deviation unless otherwise stated.

*ARB* angiotensin II receptor blocker, *CI* confidence interval, *NT-proBNP* N-terminal pro-B-type natriuretic peptide, *UACR* urine albumin-to-creatinine ratio.

**Supplementary Table 7**. PAC, PRA, and ARR (full analysis set and per-protocol set)

|  | **Full analysis set** | | | | **Per-protocol set** | | | |
| --- | --- | --- | --- | --- | --- | --- | --- | --- |
|  | **Esaxerenone** | | **ARB** | | **Esaxerenone** | | **ARB** | |
|  | ***n*** | **Mean ± SD** | ***n*** | **Mean ± SD** | ***n*** | **Mean ± SD** | ***n*** | **Mean ± SD** |
| PAC, pg/mL |  |  |  |  |  |  |  |  |
| BL | 202 | 35.3 ± 24.5 | 200 | 33.9 ± 26.2 | 185 | 36.1 ± 24.9 | 180 | 34.8 ± 26.5 |
| Week 12 | 186 | 68.8 ± 47.5 | 191 | 23.5 ± 20.6 | 171 | 69.5 ± 47.8 | 172 | 23.4 ± 20.6 |
| Change from BL | 186 | 33.2 ± 39.0 | 191 | −11.0 ± 20.7 | 171 | 33.3 ± 39.0 | 172 | −11.9 ± 21.0 |
| PRA, ng/mL/h |  |  |  |  |  |  |  |  |
| BL | 202 | 0.74 ± 0.72 | 200 | 0.70 ± 0.76 | 185 | 0.73 ± 0.74 | 180 | 0.71 ± 0.78 |
| Week 12 | 186 | 1.66 ± 1.48 | 190 | 2.92 ± 6.30 | 171 | 1.64 ± 1.51 | 171 | 3.04 ± 6.58 |
| Change from BL | 186 | 0.93 ± 1.23 | 190 | 2.23 ± 5.87 | 171 | 0.92 ± 1.26 | 171 | 2.33 ± 6.15 |
| ARR |  |  |  |  |  |  |  |  |
| BL | 198 | 93.3 ± 102.8 | 198 | 81.1 ± 82.9 | 181 | 97.2 ± 105.5 | 178 | 83.3 ± 85.0 |
| Week 12 | 186 | 85.1 ± 111.7 | 189 | 35.6 ± 58.4 | 171 | 88.1 ± 115.0 | 170 | 35.7 ± 60.5 |
| Change from BL | 183 | −8.7 ± 73.4 | 187 | −48.2 ± 68.4 | 168 | −9.1 ± 76.3 | 168 | −50.2 ± 70.3 |

*ARB* angiotensin II receptor blocker, *ARR* aldosterone–renin ratio, *BL* baseline, *PAC* plasma aldosterone concentration, *PRA* plasma renin activity, *SD* standard deviation.

**Supplementary Table 8.** Changes in eGFR_creat_ and serum potassium (safety analysis set)

|  | **Esaxerenone** | | **ARB** | |
| --- | --- | --- | --- | --- |
|  | ***n*** | **Mean ± SD** | ***n*** | **Mean ± SD** |
| eGFR_creat_, mL/min/1.73 m^2^ |  |  |  |  |
| Baseline | 206 | 69.5 ± 13.1 | 208 | 69.6 ± 15.3 |
| Week 4 | 200 | 65.6 ± 13.9 | 197 | 68.8 ± 14.5 |
| Change from baseline | 199 | −3.8 ± 10.5 | 197 | −1.1 ± 7.4 |
| Week 8 | 194 | 64.1 ± 11.9 | 193 | 68.9 ± 15.5 |
| Change from baseline | 193 | −5.1 ± 7.1 | 193 | −0.8 ± 8.1 |
| Week 12 | 188 | 65.1 ± 12.7 | 192 | 69.3 ± 15.8 |
| Change from baseline | 187 | −4.4 ± 7.4 | 192 | −0.5 ± 7.9 |
| EOT | 198 | 65.0 ± 12.5 | 197 | 69.4 ± 15.8 |
| Change from baseline | 197 | −4.5 ± 7.4 | 197 | −0.5 ± 7.8 |
| Serum potassium, mEq/mL |  |  |  |  |
| Baseline | 206 | 4.07 ± 0.38 | 207 | 4.05 ± 0.40 |
| Week 2 | 201 | 4.36 ± 0.37 | 195 | 4.20 ± 0.34 |
| Change from baseline | 201 | 0.29 ± 0.36 | 194 | 0.18 ± 0.38 |
| Week 4 | 200 | 4.33 ± 0.35 | 195 | 4.21 ± 0.32 |
| Change from baseline | 200 | 0.27 ± 0.38 | 194 | 0.20 ± 0.38 |
| Week 8 | 194 | 4.34 ± 0.36 | 192 | 4.22 ± 0.35 |
| Change from baseline | 194 | 0.26 ± 0.36 | 191 | 0.20 ± 0.40 |
| Week 12 | 187 | 4.29 ± 0.42 | 192 | 4.12 ± 0.39 |
| Change from baseline | 187 | 0.23 ± 0.40 | 191 | 0.11 ± 0.39 |
| EOT | 198 | 4.29 ± 0.41 | 197 | 4.13 ± 0.39 |
| Change from baseline | 198 | 0.22 ± 0.40 | 196 | 0.11 ± 0.39 |

*ARB* angiotensin II receptor blocker, *eGFR_creat_* estimated glomerular filtration rate (creatinine-based), *EOT* end of treatment, *SD* standard deviation.

**Supplementary Table 9.** Incidence of serum potassium level < 3.5, ≥ 5.5, and ≥ 6.0 mEq/L (safety analysis set)

| **Serum potassium level** | **Esaxerenone**  ***n* = 204** | **ARB**  ***n* = 200** |
| --- | --- | --- |
| Serum potassium < 3.5 mEq/L | 4 (2.0) [0.5, 4.9] | 10 (5.0) [2.4, 9.0] |
| Serum potassium ≥ 5.5 mEq/L | 1 (0.5) [0.0, 2.7]^1^ | 0 (0.0) [0.0, 1.8] |
| Serum potassium ≥ 6.0 mEq/L | 1 (0.5) [0.0, 2.7]^1^ | 0 (0.0) [0.0, 1.8] |

Data are *n* (%) [95% CI].

^1^The single patient with serum potassium ≥ 6.0 mEq/L is also included in the ≥ 5.5 mEq/L category; therefore, the two percentages (0.5 % each) represent the same individual.

*ARB* angiotensin II receptor blocker, *CI* confidence interval.
